# *Mesona Chinensis* Benth extract prevents AGE formation and protein oxidation against fructose-induced protein glycation *in vitro*

**DOI:** 10.1186/1472-6882-14-130

**Published:** 2014-04-07

**Authors:** Sirichai Adisakwattana, Thavaree Thilavech, Charoonsri Chusak

**Affiliations:** 1 Department of Nutrition and Dietetics, Faculty of Allied Health Sciences, Chulalongkorn University, Bangkok 10330, Thailand; 2 Research Group of Herbal Medicine for Prevention and Therapeutic of Metabolic diseases, Chulalongkorn University, Bangkok 10330, Thailand; 3 Program in Biomedical Sciences, Graduate School, Chulalongkorn University, Bangkok 10330, Thailand

**Keywords:** *Mesona chinensis*, Protein glycation, Fructose, Advanced glycation end products

## Abstract

**Background:**

*Mesona chinensis* Benth (Chinese Mesona), an economically significant agricultural plant, is the most widely consumed as an herbal beverage in Southeast Asia and China. The objective of this study was to evaluate the inhibitory activity of *Mesona chinensis* (MC) extract on the formation of advanced glycation end products (AGEs) and protein oxidation in an *in vitro* model of fructose-mediated protein glycation.

**Methods:**

The content of total polyphenolic compounds was measured by using Folin–Ciocalteu assay. Antiglycation activity was determined using the formation of AGE fluorescence intensity, N^ϵ^-(carboxymethyl)lysine (CML), the level of fructosamine, and the formation of amyloid cross β-structure. The protein oxidation was examined using the level of protein carbonyl content and thiol group.

**Results:**

Our results revealed that the content of total polyphenolic compound in MC extract was 212.4 ± 5.6 mg gallic acid equivalents/g dried extract. MC extract (0.25-1.00 mg/mL) significantly inhibited the formation of fluorescence AGEs in fructose-glycated bovine serum albumin (BSA) during 4 weeks of study. Furthermore, MC extract also decreased the level of N^ϵ^-CML, fructosamine, and amyloid cross β-structure in fructose-glycated BSA. While the total thiol group was elevated and the protein carbonyl content was decreased in BSA incubated with fructose and MC extract.

**Conclusions:**

The extract of MC inhibits fructose-mediated protein glycation and protein oxidation. This edible plant could be a natural rich source of antiglycation agent for preventing AGE-mediated diabetic complication.

## Background

Chronic hyperglycemia plays a vital role in the development of long-term diabetic complications by increasing non-enzymatic protein glycation and the gradual formation of advanced glycation end products (AGEs) in body tissues [1]. Protein glycation causes marked changes in the structural properties and stability that impair protein function associated with the pathogenesis of age-related diseases [2]. Consequently, the interaction of AGEs with receptors for AGEs (RAGE) directly activates multiple intracellular signaling, gene expression, and the secretory pro-inflammatory molecules accompanied by increasing free radicals that contribute towards pathologic complications related to diabetes such as atherosclerosis, nephropathy, peripheral neuropathy, retinopathy and cataract [3]. Aminoguanidine, a prototype therapeutic agent for inhibition of AGE formation, has received the most interest from a clinical trials perspective [4,5]. However, it may have serious toxicity when administered for diabetic nephropathy [5]. Therefore, there is considerable interest in search of plant-based diets with antiglycation activity as they may potentially inhibit AGE formation resulting in delaying and preventing the onset of diabetic complications with minimal side effects [6-8].

Many studies have shown the beneficial effect of plant-based diets for the inhibition of protein glycation *in vitro* and *in vivo*[9,10]. *Mesona chinensis* Benth (Chinese Mesona), belonging to the Lamiaceae family, is an economically important agricultural plant in Southeast Asia and China [11]. This plant-based diet is the most widely consumed as an herbal beverage and a gelatin-type dessert (Grass jelly). In addition, it has been utilized in ancient folk medicine for treatment of hypertension, diabetes, and liver diseases [10]. The assessment of available nutrient status has shown that *M. chinensis* (MC) includes 17 amino acids (7 essential amino acids), carbohydrate, fat, fiber, polyphenols, and flavonoids [12,13]. However, there were no reports in the scientific literature on antiglycation activity of MC. Hence, the aim of this study were carried out to investigate the inhibitory effects of MC against fructose-mediated non-enzymatic glycation and oxidation-dependent damages to BSA. These results reported herein support the notion that this plant-based diet could be a new avenue for treatment and prevention of protein glycation and related diseases conditions.

## Methods

### Chemicals

Bovine serum albumin (BSA, fraction V), aminoguanidine hydrochloride (AG), nitroblue tetrazolium (NBT), 1-deoxy-1-morpholino-D-fructose (1-DMF), guanidine hydrochloride, congo red, 5,5'-dithiobis(2-nitrobenzoic acid) (DTNB) and L-cysteine were purchased from Sigma-Aldrich Co. (St. Louis, MO, USA). D-fructose and 2,4-dinitrophenyl hydrazine (DNPH) were purchased from Ajax Finechem (Taren Point, Australia). Trichloroacetic acid (TCA) was purchased from Merck (Darmstadt, FR, Germany). OxiSelect™ N^ϵ^-(carboxymethyl) lysine (CML) ELISA kit was obtained from Cell Biolabs (San Diego, CA, USA). All other chemicals and solvents used in this study were of analytical grade.

### Plant preparation and extraction

The dried whole plants were purchased from a specific herbal drugstore, Bangkok, Thailand. The plant has been authenticated at the Professor Kasin Suvatabhandhu Herbarium, Department of Botany, Chulalongkorn University, Thailand, Voucher specimen: A013637 (BCU). The dried plants (300 g) were boiled in distilled water (4 L) at 90°C for 4 hours. After boiling, the residue of plant was filtered with a colander. The aqueous extraction was dried using a spray dryer SD-100 (Eyela world, Tokyo Rikakikai Co., LTD, Japan). The condition of spray dryer was inlet temperature 178-180°C, outlet temperature 85-95°C, blower (0.80-0.90) m^3^/min, and atomizing at 90 kPa. The powder extract was kept in a dry place.

### Measurement of total polyphenolic content

The content of total polyphenolic compounds in the extract was determined using the Folin-Ciocalteu’s phenol reagent [14]. The content of total polyphenolic compounds was expressed as mg gallic acid equivalents/g dried extract.

### *In vitro* glycation of bovine serum albumin

Glycated BSA was done according to a previously published method [15]. Briefly, 10 mg/mL BSA (0.50 mL) was incubated with 0.46 mL of 500 mM fructose in 100 mM phosphate buffered-saline (pH 7.4) containing 0.02% sodium azide at 37°C for 4 weeks. Before incubation, 0.04 mL of MC extract (final concentration: 0.25-1.00 mg/mL) and AG (final concentration: 1.00 mg/mL) were added into the reaction mixtures. The formation of fluorescent AGEs was measured by using a spectrofluorometer. The fluorescent intensity was measured at an excitation wavelength of 355 nm and emission wavelength of 460 nm. The percentage of fluorescent AGE formation was calculated as follows:

$$\begin{array}{l} \text{Inhibition}\;\text{of}\;\text{fluorescent}\;\text{AGEs}\;\left(\%\right)\\ \;=\left[\left(F_{C}-F_{\mathrm{CB}}\right)-\left(F_{S}-F_{\mathrm{SB}}\right)/\left(F_{C}-F_{\text{CB}}\right)\right]\;x100 \end{array}$$

Where F_C_ and F_CB_ were the fluorescent intensity of control with fructose and blank of control without fructose, F_S_ and F_SB_ were the fluorescent intensity of sample with fructose and blank of sample without fructose.

The concentration of non-fluorescent AGEs (N^ϵ^-(carboxymethyl) lysine, CML), a major non-fluorescent AGEs structure, was measured by using enzyme linked immunosorbant assay (ELISA) kit according to the manufacturer’s protocol. The concentration of N^ϵ^-CML was calculated by using a standard curve of N^ϵ^-CML-BSA.

### Determination of fructosamine

The level of fructosamine was measured by using nitroblue-tetrazolium (NBT) dye according to a previous published method [16]. Briefly, glycated BSA (10 μL) was incubated with 0.5 mM NBT (90 μL) in 100 mM carbonate buffer, pH 10.4 at 37°C. The absorbance was recorded at 530 nm. The level of fructosamine was calculated by using the different absorption at the time point of 10 and 15 min. The level of fructosamine was calculated from a standard curve (0.31-5.0 mM) prepared using 1-deoxy-1-morpholino-fructose (1-DMF).

### Determination of protein aggregation

Amyloid cross β-structure, a common marker for protein aggregation was measured by using a congo red assay according to a previous published method with minor modifications [17]. Briefly, the glycated BSA (50 μL) was incubated with 50 μL of 100 μM congo red in 10% (v/v) ethanol/PBS for 20 min at 25°C. The absorbance was measured at 530 nm.

### Determination of protein thiol groups

Protein thiol groups were measured according to a previous published method with minor modifications [18]. In brief, glycated BSA (10 μL) was reacted with 90 μL of 5 mM 5,5'-dithiobis(2-nitrobenzoic acid) (DTNB) in 100 mM phosphate buffered-saline (pH 7.4) at room temperature for 15 min. Thereafter, the absorbance was measured at 412 nm. The level of protein thiol groups was calculated from a standard curve (0.015-0.50 mM) prepared using L-cysteine. The results were expressed as nmol L-cysteine/mg protein.

### Determination of protein carbonyl contents

The protein carbonyl contents were measured according to a previous published method with minor modifications [18]. In brief, glycated BSA (0.10 mL) was incubated with 0.40 mL of 10 mM 2,4-dinitrophenylhydrazine (DNPH) in 2.5 M HCl at room temperature for 60 min. Subsequently, glycated BSA was precipitated by 0.50 mL of 20% (w/v) trichloroacetic acid (TCA), left on ice for 5 min, and centrifuged at 10,000 g at 4°C for 10 min. The pellet was washed three times using 1:1 (v/v) ethanol:ethyl acetate mixture (1 mL). The final pellet was dissolved in 6 M guanidine hydrochloride (0.25 mL). The absorbance was recorded at 370 nm. The level of protein carbonyl contents was calculated by using an absorption coefficient of 22,000 M^-1^ cm^-1^. The results were expressed as nmol carbonyls/mg protein.

### Statistical analysis

Data were expressed as mean ± standard error of mean (SEM) (*n* = 3). The statistical significance of the results was evaluated by using one-way ANOVA. The Tukey’s HSD test was used to analyze the statistically significant differences of mean. *P* < 0.05 was considered to be statistically significant.

## Results

### Phytochemical analysis of MC extract

The present data showed that the total polyphenolic content of MC extract was 212.4 ± 5.6 mg gallic acid equivalents/g dried extract.

### The effects of MC extract on the formation of fluorescence AGEs and N^ϵ^-CML

**Figure 1:**
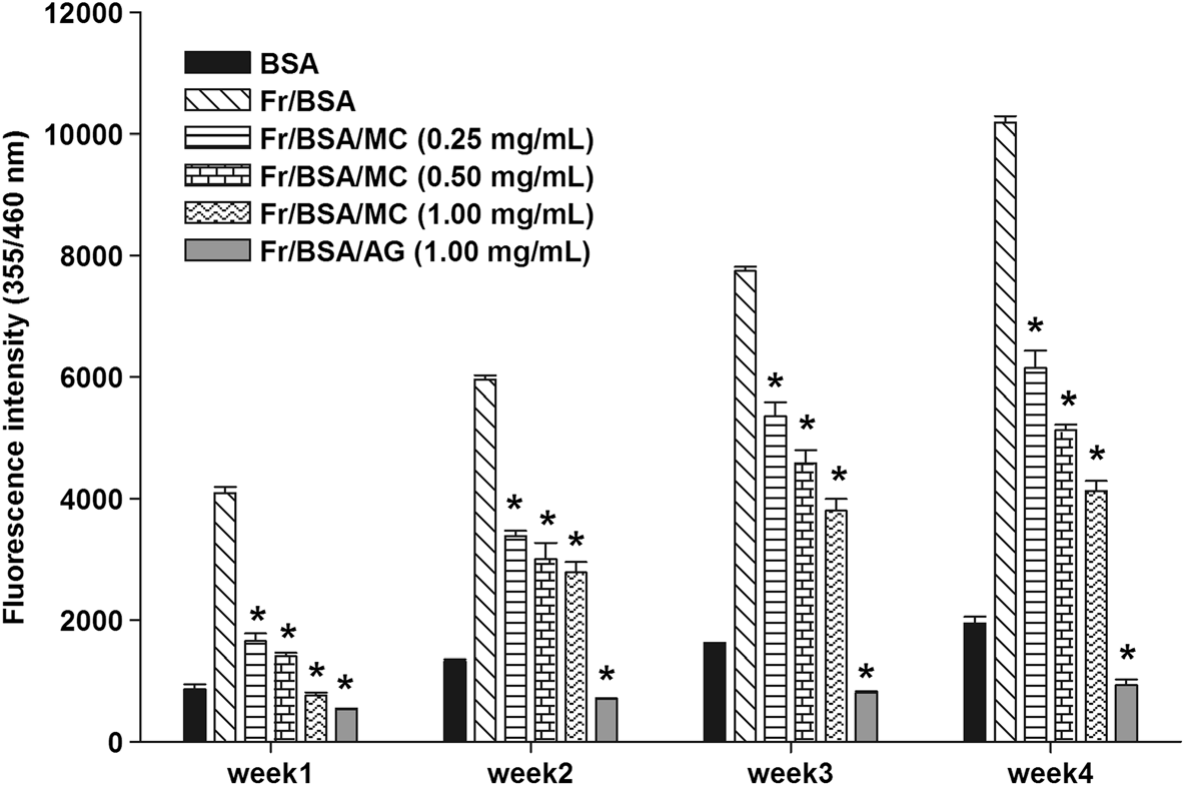
**The effects of MC extract on the formation of fluorescent AGEs in BSA incubated with fructose.** Each value represents the mean ± SEM (n = 3). *P < 0.05 when compared to BSA/fructose at the same week.

As shown in Figure 1, the formation of AGEs was observed weekly by the measurement of increasing fluorescent intensity in fructose-glycated BSA. The significant increase in fluorescent intensity in BSA incubated with fructose was seen during 4 weeks of the incubation. The results demonstrated that addition of MC extract (0.25-1.00 mg/mL) into the solution significantly reduced the formation of fluorescent AGE in a concentration-dependent manner throughout the study period. Similar to the effect of MC (1.00 mg/mL), a significant inhibition of fluorescent AGEs was observed in fructose-glycated BSA plus AG (1.00 mg/mL). The percentage inhibition of MC (0.25-1.00 mg/mL) is shown in Table 1. At week 4 of incubation, the percentage inhibition of MC extract (0.25-1.00 mg/mL) was approximately 39.60-59.42%, whereas AG (1.00 mg/mL) decreased the formation of fluorescence AGEs by 90.84%. The results indicated that MC extract had 1.5-times less potency than AG.

**Table 1:** The percentage inhibition of MC extract on the formation of fluorescent AGEs during the experimental period

**Samples % Inhibition**				
	**Week 1**	**Week 2**	**Week 3**	**Week 4**
Fr/BSA/MC 0.25 mg/mL	59.65 ± 3.22	43.14 ± 1.66	30.78 ± 3.01	39.60 ± 2.96
Fr/BSA/MC 0.50 mg/mL	65.80 ± 1.27	49.44 ± 5.04	40.81 ± 2.39	49.61 ± 1.18
Fr/BSA/MC 1.00 mg/mL	81.39 ± 1.23	53.43 ± 2.40	50.83 ± 2.85	59.42± 1.78
Fr/BSA/AG 1.00 mg/mL	86.81 ± 0.36	88.27 ± 0.35	89.51± 0.27	90.84 ± 0.88

Data are expressed as mean ± SEM, n = 3 (Fr: fructose; MC: *Mesona chinensis*; AG: aminoguanidine).

**Figure 2:**
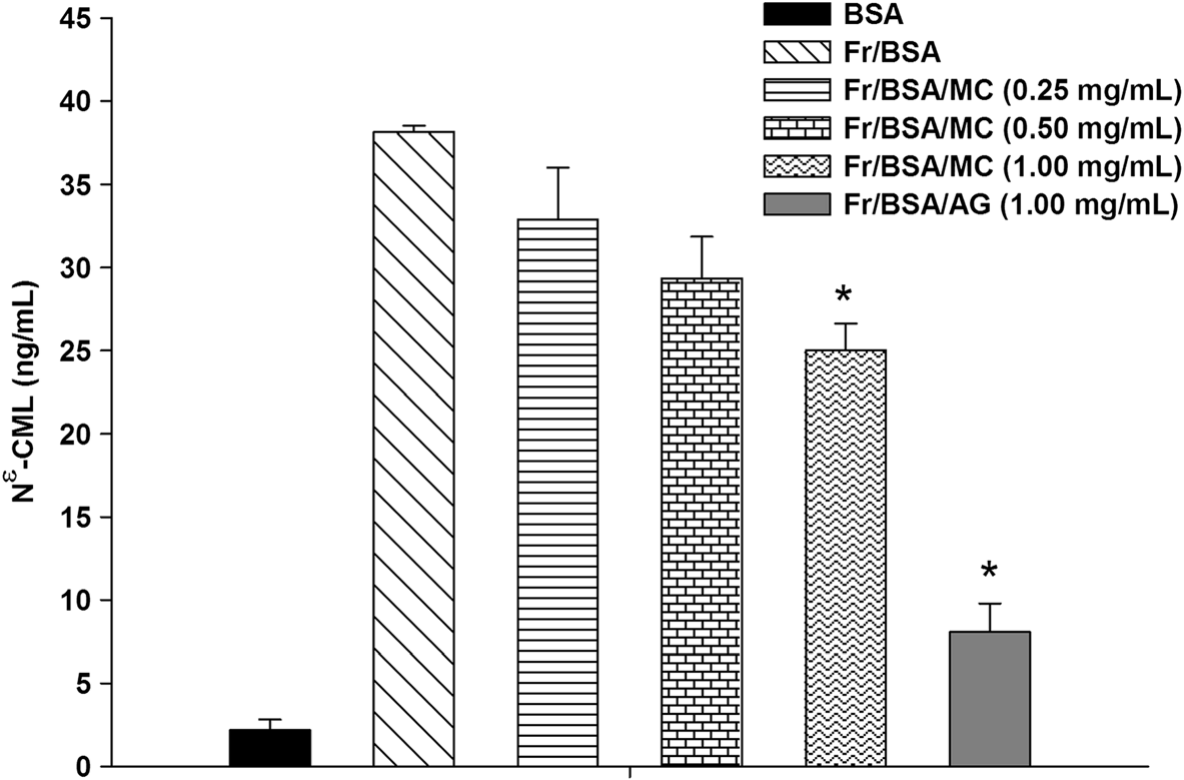
**The effects of MC extract on the level of N**^**ϵ**^**-(carboxymethyl) lysine (CML) in BSA incubated with fructose after 4 weeks of incubation.** Each value represents the mean ± SEM (n = 3). *P < 0.05 when compared to BSA/fructose.

The levels of N^ϵ^-CML, a biomarker for the formation of non-fluorescent AGE are shown in Figure 2. According to the results, MC extract (1.00 mg/mL) and AG (1.00 mg/mL) decreased 26.0% and 59.7% of N^ϵ^-CML formation as compared to fructose-glycated BSA, suggesting that MC extract had 2.3-times less potency than AG.

### The effects of MC extract on the level of fructosamine

**Figure 3:**
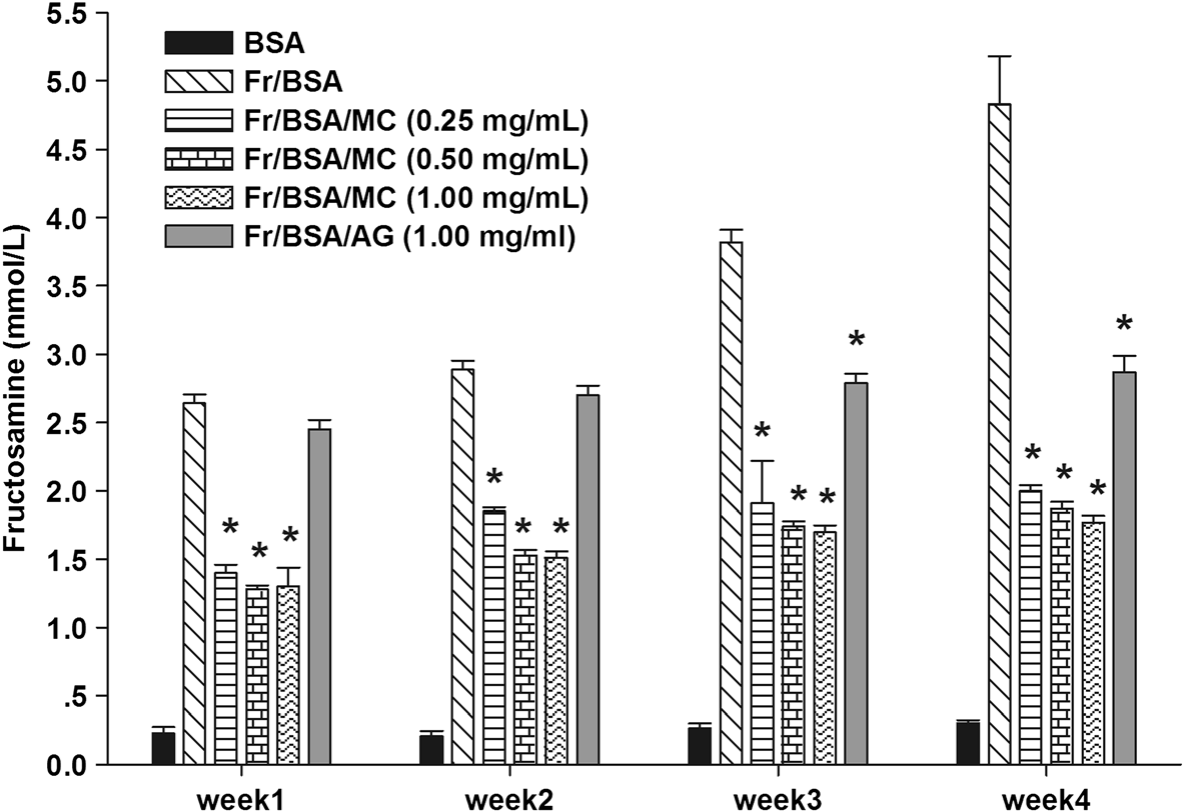
**The effects of MC extract on the level of fructosamine in BSA incubated with fructose.** Each value represents the mean ± SEM (n = 3). *P < 0.05 when compared to BSA/fructose at the same week.

The effects of MC extract on the level of fructosamine are presented in Figure 3. The level of fructosamine in fructose-glycated BSA markedly increased throughout 4 weeks of the experiment. The increasing level of fructosamine was attenuated by MC extract during 4 weeks of study. In comparison at the end of study, the percentage inhibition of MC extract (0.25-1.00 mg/mL) was approximately 58.6-63.3%, whereas AG (1.00 mg/mL decreased the level of fructosamine by 40.6%.

### The effects of MC extract on the level of amyloid cross-β structure

**Figure 4:**
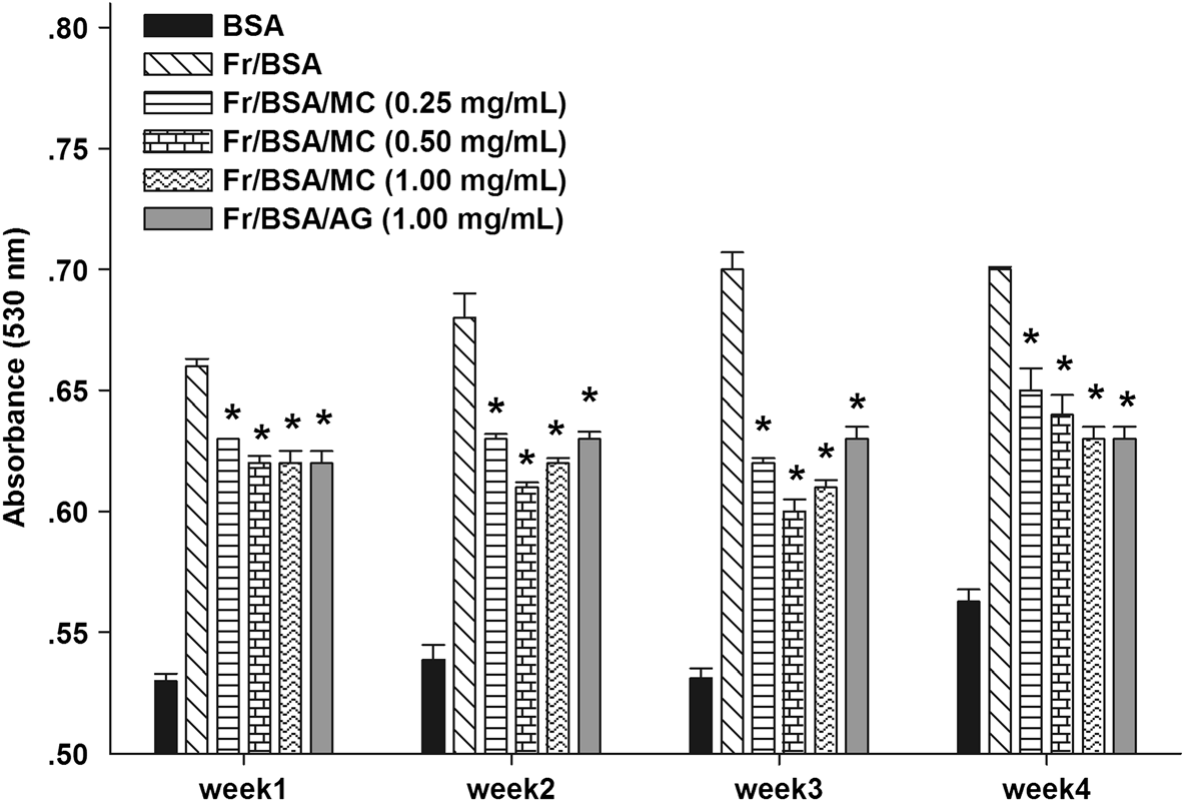
**The effects of MC extract on the formation of amyloid cross-β structure in BSA incubated with fructose.** Each value represents the mean ± SEM (n = 3). *P < 0.05 when compared to BSA/fructose at the same week.

Congo red assay is generally used to measure the amount of a protein modification called amyloid cross-β structure in glycated BSA. As shown in Figure 4, BSA incubated with fructose elevated the level of amyloid cross-β conformation throughout 4 weeks of experimental period. In the meanwhile, fructose-glycated BSA incubated with MC extract (0.25-1.00 mg/mL) significantly attenuated an increase in the level of amyloid cross-β structure. At 4 week of incubation, MC extract at concentrations of 0.25-1.00 mg/mL reduced the level of amyloid cross-β structure in a concentration-dependent manner (8.1%, 9.7% and 10.3%) Similarly, a significant decrease in the level of amyloid cross-β structure (10.1%) was observed in the presence of AG (1 mg/mL) at week 4 of incubation.

### The effects of MC on protein oxidation

**Figure 5:**
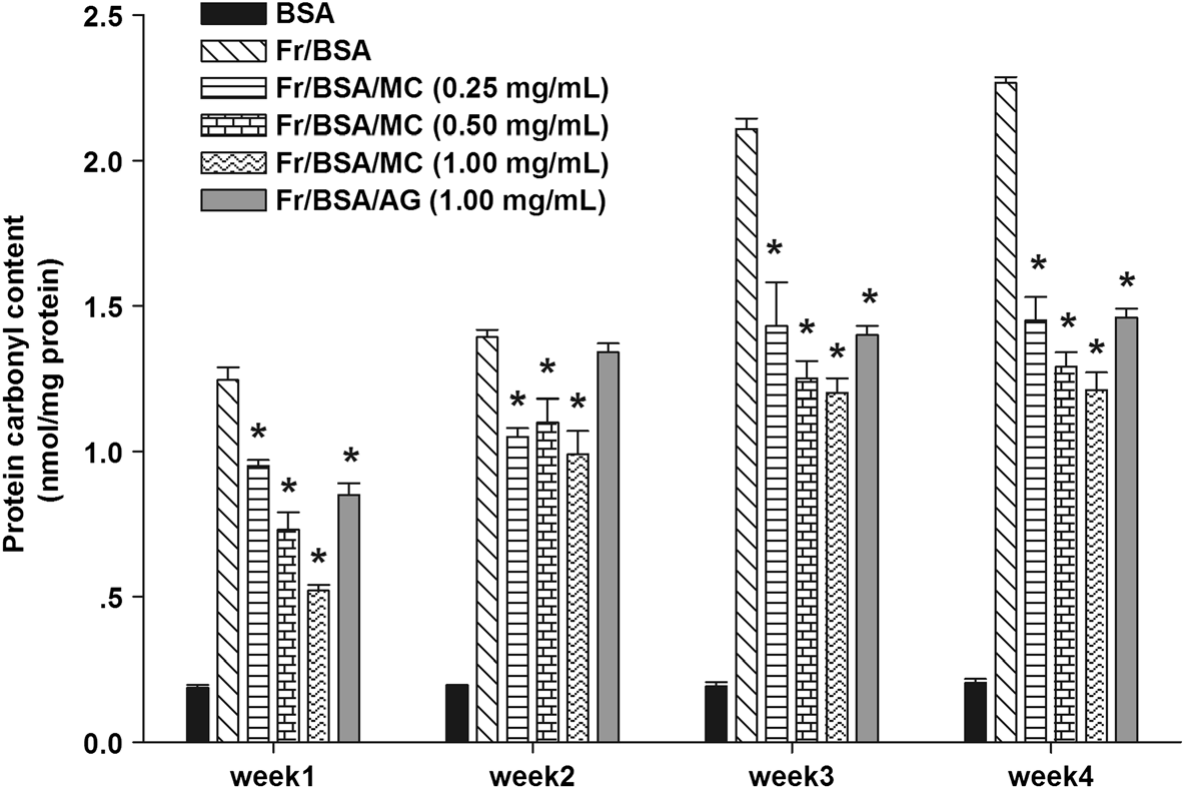
**The effects of MC extract on the protein carbonyl content in BSA incubated with fructose.** Each value represents the mean ± SEM (n = 3). *P < 0.05 when compared to BSA/fructose at the same week.

In order to access the protein oxidation mediated by glycation process, the level of carbonyl content and thiol groups was used for determination. As shown in Figure 5, the carbonyl content of fructose-glycated BSA was significantly increased during the experimental period, whereas MC extract (0.25-1.00 mg/mL) significantly suppressed an increase in protein carbonyl content of fructose-glycated BSA. When comparing with fructose-glycated BSA at week 4, the percentage reduction of carbonyl content by MC extract (0.50-1.00 mg/mL) ranged from 36.2 to 46.7%. A significant reduction of protein carbonyl content (24.4%) was seen in the presence of AG (1 mg/mL) at the same week.

**Figure 6:**
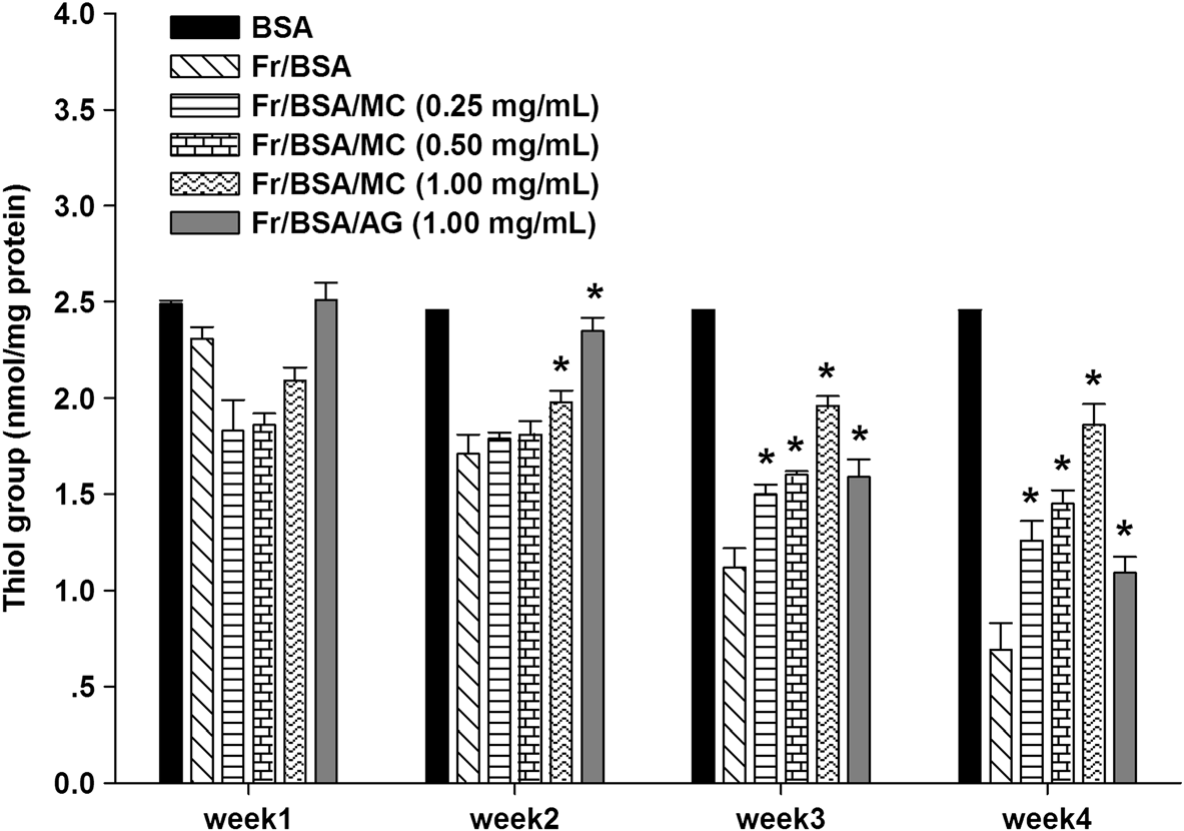
**The effects of MC extract on the level of thiol group in BSA incubated with fructose.** Each value represents the mean ± SEM (n = 3). *P < 0.05 when compared to BSA/fructose at the same week.

The effects of MC extract on the oxidation of protein thiols are presented in Figure 6. When BSA was incubated with fructose, the level of thiol groups had continuously declined throughout the experimental period. Interestingly, there was a significant increase in the level of thiol groups after addition of MC extract (0.25-1.00 mg/mL) as well as AG (1.00 mg/mL). The findings showed that the percentage prevention of thiol group by MC ranged from 46.2 to 64.1%, whereas AG (1 mg/mL) significantly prevented (57.9%) the depletion of protein thiol groups at the week 4 of incubation. According to the results, MC extract is more potent in the reduction of fructose-induced protein oxidation than AG.

## Discussion

Glycated proteins are commonly formed by a non-enzymatic reaction between the interaction of reducing sugars (fructose and glucose) and amino group of protein through a nucleophilic addition with formation of Schiff bases [1]. The unstable Schiff bases further rearrange to produce the formation of reversible Amadori products (such as fructosamine). Subsequently, the Amadori products further form cross-linked structures termed AGEs which can be classified into two major groups: fluorescent and crosslinking structures (pentosidine, crosslines, and imidazolones) and non-fluorescent and non-crosslinking structures (N^ϵ^-CML). The formation of AGEs occurs through multiple processes related in the part to generate reactive oxygen species (ROS). It is noteworthy that the superoxide anion productions (1,2- and 2,3-enolization of the Schiff's base and oxidation of the enolate anion) are particularly generated from early glycation products [19,20]. Consequently, reactive oxygen species-mediated reactions cause the structural fragmentation to create the short-chain carbohydrate intermediates, which then alters sequentially with lysine and arginine residues to produce AGEs [19,20]. Currently, the possible anti-glycation mechanisms have been proposed such as breaking the cross-linking structures in the formed AGEs, blocking the carbonyl or dicarbonyl groups in reducing sugars, Schiff bases or Amadori products, and inhibiting the formation of late-stage Amadori products [21]. It has revealed that aminoguanidine acts as a carbonyl trapping agent by forming guanidine-dicarbonyl adducts, thereby reducing the numbers of free carbonyl groups in reducing sugars during the early stages of glycation [21,22].

There has been research into the role of dietary fructose in the development of diabetes complications. Excessive fructose consumption has been linked to the development of metabolic syndromes and obesity [23]. It is well documented that long-term fructose consumption leads to glycoxidation and generation of ROS causing oxidative damage and cellular dysfunction that accompany the aging process [24]. The concentration of fructose in human tissues and fluids is generally much lower than that of glucose. Under hyperglycemic conditions, fructose can be synthesized by oxidation of sorbitol through a reaction catalyzed by polyol dehydrogenase. Ocular lens are one of specific organs where the sorbitol pathway is active, fructose accumulates to levels higher than in blood circulation. These results lead to accelerate the reaction between fructose and protein, giving AGE formation and accumulation in len proteins. From previous studies comparing the reactivity of glucose and fructose with proteins at physiological temperature and the equal concentration, a faster accumulation of protein-bound fluorescence and cross-linking products was from fructose [18,25]. In addition, fructose has been shown to be more rapidly produced dicarbonyl compounds and hydroxyl radicals by autoxidation, compared to glucose [26]. Currently, a study has been published on the site specificity of modification of lysine residues and total of N^ϵ^-CML in BSA incubated with fructose and glucose. The major site of modification by fructose, like glucose, is Lysine-524 and 31 and 76% loss of the corresponding unmodified tryptic peptide is observed at Gln525-Lys533 [22]. The fructose-modified BSA has the yield of N^ϵ^-CML being up to 17-fold higher than glucose-modified BSA.

In a present study, we investigated the influence of MC extract on the formation of total AGEs. The results showed that MC extract efficiently inhibited fluorescent and non-fluorescent AGE formation. Furthermore, it also reduced the level of fructosamine and amyloid cross β-structure in fructose-glycated BSA. A significant increase of protein carbonyl content and oxidation of thiols in BSA were seen when the protein was glycated by fructose. When MC extract was added to the same systems, it significantly suppressed these processes. Our findings indicate that MC extract has high content of polyphenolic compounds. Several major mechanisms by which polyphenols block the carbonyl group in reducing sugars and break the crosslinking structure in the formed AGEs have recently been proposed for antiglycation activity [21]. The reduction of free radical generation by antioxidant activity of polyphenols may highlight other mechanisms for the prevention of AGE formation [21]. Recent studies carried out over the past few years have shown that polyphenolic compounds from the edible plants may play a protective role against monosaccharide-induced protein glycation [27,28]. The earlier studies report a strong correlation between the polyphenolic content in the tested plant extracts and the ability to inhibit protein glycation [29,30]. Our previous studies have previously shown the inhibitory activity on edible plant extracts containing polyphenolic compounds [7,31]. According to the results obtained, we addressed the hypothesis that polyphenolic compounds in the extract may be a major contributor to inhibit the formation of AGEs. However, certain active biological constituents of MC extract remain unknown. To prove this hypothesis, separation and characterization of polyphenolic compound in MC extract using HPLC-MS are required for further study.

In general, protein glycation can directly affect the formation of protein aggregation. The insoluble aggregates can form amyloid cross β-structure leading to alter protein structure and stability [17]. The long-term accumulation of amyloid cross β-structures in the tissues may cause the progression of pancreatic islet amyloidosis which directly destroys β-cell and impairs insulin secretion [32]. The present findings demonstrate that MC extract reduces the formation of amyloid cross β-structures in BSA *in vitro*. This beneficial effect might help reduce the risk of developing diabetes complications.

Glycation with fructose rapidly induces protein oxidation and consequently alters the structure of BSA associated with modifications of its biological properties. In particular, the thiol group of Cys residues is particularly prone to oxidative attack by free radical damage to proteins, as it is known that the formation of disulfide bonds occurs in protein aggregation and results in the loss of enzymatic activity [33]. The direct oxidation of amino acid (Lys, Arg, Thr) or secondary reaction of amino acid residues (Cys and His) with reactive carbonyl compounds can produce the formation of protein carbonyl derivatives [34]. Our findings are consistent with previous literatures indicating that fructose-induced glycation increased protein oxidation as evidenced by decreased thiol group and increased protein carbonyl content [16]. The present findings suggest that the MC has shown remarkable potential in protecting the protein thiols and reducing the protein carbonyl content. There is a well-documented evidence for antioxidant activity of *Mesona procumbens* Hemsley (Hsian-tsao), the same genus of *M. chinensis*[35]. The crude extract of *M. procumbens* leaf gum has also shown a remarkably high scavenging activity toward chemically generated reactive oxygen species [36]. Recent studies in our findings have shown the antioxidant activity of MC extract tested by different methods [37]. The findings indicated that MC extract had 7-times less potency than ascorbic acid. On the DPPH assay, MC extract had 7-times less potency than ascorbic acid. In the meanwhile, MC extract had 3-times and 3.6-times less potency of hydroxyl and superoxide radical scavenging activity than trolox, respectively. According to the abovementioned antiglycation mechanisms, MC extract may inhibit AGE formation by scavenging free radicals formed *in vitro* by auto-oxidation of sugars and/or oxidative degradation of Amadori products. Most antiglycation agents with antioxidant activity from the edible plants have been reported to possess polyphenolic compounds [21,28]. It has revealed that the inhibitory activity of polyphenols against protein glycation was strongly related to their scavenging effect on free radicals derived from the glycoxidation process [38]. According to many supporting documents, it can be assumed that polyphenolic compounds in MC extract may contribute to the antioxidant activity and antiglycation.

## Conclusion

MC extract has potent inhibitory effects on fructose-induced protein glycation and oxidation damage in BSA. MC extract with high antiglycation activity may offer remarkable prospects for the preventive treatment of AGE-mediated diabetic complications. For these reasons, further studies should focus on the outcome of investigating effects in animal models.

## Abbreviations

AGEs: Advanced glycation end products; Nϵ-CML: N^ϵ^-(carboxymethyl) lysine; BSA: Bovine serum albumin; AG: Aminoguanidine; MC: *Mesona chinensis*.

## Competing interests

The authors declare that they have no competing interests.

## Authors’ contributions

SA was responsible for conception and design, drafted the manuscript and revised it critically for important intellectual content. TT and CC conducted the experiments, organized the data analysis, and interpretation of data. All authors contributed to the drafting of the manuscript and agreed on the final version of the manuscript.

## Pre-publication history

The pre-publication history for this paper can be accessed here:

http://www.biomedcentral.com/1472-6882/14/130/prepub
